# The mutation landscape of multiple cancer predisposition genes in Chinese familial/hereditary breast cancer families

**DOI:** 10.20892/j.issn.2095-3941.2021.0011

**Published:** 2021-09-28

**Authors:** Li Dong, Hailian Zhang, Huan Zhang, Yingnan Ye, Yanan Cheng, Lijuan Li, Lijuan Wei, Lei Han, Yandong Cao, Shixia Li, Xishan Hao, Juntian Liu, Jinpu Yu

**Affiliations:** 1 Cancer Molecular Diagnostics Core, Tianjin Medical University Cancer Institute and Hospital, National Clinical Research Center for Cancer, Key Laboratory of Cancer Prevention and Therapy, Tianjin, Tianjin’s Clinical Research Center for Cancer, Key Laboratory of Breast Cancer Prevention and Therapy, Tianjin Medical University, Ministry of Education, Tianjin 300060, China; 2 The Second Department of Breast Cancer, Tianjin Medical University Cancer Institute and Hospital, National Clinical Research Center for Cancer, Key Laboratory of Cancer Prevention and Therapy, Tianjin, Tianjin’s Clinical Research Center for Cancer, Key Laboratory of Breast Cancer Prevention and Therapy, Tianjin Medical University, Ministry of Education, Tianjin 300060, China; 3 Cancer Prevention Center, Tianjin Medical University Cancer Institute and Hospital, National Clinical Research Center for Cancer, Key Laboratory of Cancer Prevention and Therapy, Tianjin, Tianjin’s Clinical Research Center for Cancer, Key Laboratory of Breast Cancer Prevention and Therapy, Tianjin Medical University, Ministry of Education, Tianjin 300060, China; 4 Department of Oncology, Tianjin Third Central Hospital, Tianjin Institute of Hepatobiliary Disease, Tianjin Key Laboratory of Artificial Cell, Artificial Cell Engineering Technology Research Center of Public Health Ministry, Tianjin 300170, China; 5 Analyses Technology Co. Ltd., Beijing 102600, China

**Keywords:** Familial breast cancer, predisposition genes, DNA damage repair genes, clinical features

## Abstract

**Objective:**

Approximately 5%–10% of breast cancer (BC) patients display familial traits that are genetically inherited among the members of a family. The purpose of this study was to profile the germline mutations in 43 genes with different penetration rates and their correlations with phenotypic traits in Chinese familial BC families.

**Methods:**

Ion Torrent S5™-based next generation sequencing was conducted on 116 subjects from 27 Chinese familial BC families.

**Results:**

Eighty-one germline mutations in 27 BC predisposition genes were identified in 82.8% (96/116) of the cases. Among these, 80.8% of the mutated genes were related to DNA damage repair. Fourteen possible disease-causing variants were identified in 13 of 27 BC families. Only 25.9% (7/27) of the BC families exhibited hereditary deficiency in *BRCA1/2* genes, while 22.2% of the BC families exhibited defects in *non-BRCA* genes. In all, 41.7% (40/96) of the mutation carriers had *BRCA* mutations, 88.5% (85/96) had *non-BRCA* mutations, and 30.2% (29/96) had both *BRCA* and *non-BRCA* mutations. The BC patients with *BRCA* mutations had a higher risk of axillary lymph node metastases than those without mutations (*P* < 0.05). However, the BC patients with *non-BRCA* mutations frequently had a higher occurrence of benign breast diseases than those without mutations (*P* < 0.05).

**Conclusions:**

In addition to *BRCA1/2*, genetic variants in *non-BRCA* DNA repair genes might play significant roles in the development of familial/hereditary BC. Therefore, profiling of multiple BC predisposition genes should be more valuable for screening potential pathogenic germline mutations in Chinese familial/hereditary BC.

## Introduction

For Chinese women, breast cancer (BC) has become the most common malignant tumor and the fifth most common cause of cancer death. Approximately 5%–10% of BC patients display familial traits that are genetically inherited among the members of a family^1^, and are significantly regulated by varied genetic factors. Among these genetic factors, driver genes directly stimulate BC carcinogenesis, while predisposition genes generally increase the hereditary genetic risk of BC and are the most important causes of familial clustering in BC. However, no study focusing on the germline genetic profiling of multiple BC predisposition genes has been reported in Chinese hereditary BC families. *BRCA1* and *BRCA2* are 2 well-known high penetration BC predisposition genes in hereditary BC^2^. *BRCA1/2* mutations are characteristic of an increased lifetime risk for hereditary breast and ovarian cancer syndrome^3^. The cumulative risk of BC in women with *BRCA* mutations is as high as 80% by the age of 70^4,5^. Clinical studies have shown that patients with *BRCA* mutations have a higher incidence of early-onset BC, bilateral BC, triple-negative BC, lymph node metastasis, and ipsilateral and contralateral BC recurrence^6–9^. Patients with *BRCA*-related BC are also at high risk for other cancers, such as pancreatic cancers, gastrointestinal malignancies, and melanomas^10^. Identification of germline mutations in *BRCA1/2* will not only help to identify high risk hereditary BC patients, but will also change screening, cancer risk management, and therapeutic strategies for their family members.

However, only 20%–40% of familial hereditary BC are caused by *BRCA1/2* mutations^11^. There is a large percentage of familial BC not associated with *BRCA1/2* mutations. Currently, more BC predisposition genes have been identified, including genes with high penetration (*TP53*, *CDH1*, *PTEN*, and *STK11*), moderate penetration (*PALB2*, *CHEK2*, *ATM*, *NBN*, etc.), and low penetration (*MLH1*, *MSH2*, *MSH6*, *PMS2*, *MEN1*, etc.)^12–14^. Most BC predisposition genes are DNA damage repair (DDR)-related genes. DDR is an important part of the mammalian cell defense mechanism and includes 5 different but functionally interrelated pathways: base excision repair (BER), nucleotide excision repair, mismatch repair (MMR), homologous recombination repair (HR), and nonhomologous end joining^15^. DDR genes recover the DNA damage caused by various factors *in vivo* and *in vitro*, thus maximizing the stability of genetic material. The decline or lack of DDR ability can lead to genome instability and the occurrence of cancer^16^.

It has been reported that genomic instability caused by DDR gene deficiency is one of the most important reasons for the occurrence of BC^17–19^. Comprehensive screening of genetic variants of DDR genes would therefore help to precisely evaluate hereditary susceptibilities to BC in high risk families. Next-generation sequencing (NGS) has recently enabled massive parallel sequencing at low cost, which makes high-throughput gene testing commercially available for hereditary BC susceptibility assessment with high accuracy and high efficiency^20^.

In this study, a total of 116 subjects from 27 Chinese hereditary BC families were enrolled, including both BC patients and their relatives. Ion Torrent S5™-based NGS was conducted to detect multiple types of germline variants in 43 genes and compare their correlations with phenotypic traits. We found that 80.8% of the mutated genes were related to DDR. Only 25.9% of BC families exhibited hereditary deficiency in *BRCA1/2* genes, while 22.2% of the BC families exhibited defects in *non-BRCA* genes. The *BRCA* mutation patients had a higher occurrence of axillary lymph node metastases, while the *non-BRCA* mutation patients frequently had a higher occurrence of benign breast diseases than those without mutations. Genetic variants in *non-BRCA* DDR genes might therefore play significant roles in the development of Chinese familial/hereditary BC, and more extensive BC predisposition genes should be considered to evaluate hereditary BC susceptibilities in high risk families.

## Materials and methods

### Sample collection

A total of 27 hereditary BC families in China were enrolled and admitted to the Second Department of Breast Cancer of Tianjin Medical University Cancer Institute and Hospital (TMUCIH) from January 2017 to January 2019. The criterion for the collection of hereditary BC families was that the families should include ≥ 2 patients with breast and/or ovarian cancer among first- and second-degree relatives. This study was approved by the Ethics Committee of Tianjin Medical University (Approval No. Ek2018050). Written consent was obtained from all patients.

We selected at least 1 BC patient and 1 family member from each hereditary BC family. Finally, a total of 116 subjects from 27 families were collected, which included 45 patients (42 BC, 2 ovarian cancer, and 1 endometrial cancer) and 71 healthy family members. All subjects were Chinese. Among the 42 BC patients, 36 were initially treated at TMUCIH. Clinical characteristics for these 36 patients were collected, including the age of onset, unilateral/bilateral, primary tumor diameter size, regional lymph node status, clinical stage, tumor grade, histological type, luminal type, benign breast disease, and recurrence or metastasis. When the clinical characteristics of the 36 patients were analyzed, the data of the additional 47 BC patients with a family history of BC were also included. These 47 familial BC patients were all females and were also treated at TMUCIH. The age of the patients ranged from 26–76 years. The median age was 51 years and the average age was 50.1 years. Of them, 31.9% were younger than 45 years, 36.1% had lymph node metastasis, 17.0% were triple negative BC, 29.7% were in stage III, and 21.2% were histological grade III (details are shown in **Supplementary Table S1**). All 47 familial BC patients were sequenced by the same assay panel as the 27 pedigree samples.

This study was approved by the ethics committee of TMUCIH, and all included subjects signed informed consent forms.

### NGS panel

In this study, the 43 genes selected were as follows: *AKT1*, *APC*, *ATM*, *ATR*, *BAP1*, *BARD1*, *BLM*, *BRAF*, *BRCA1*, *BRCA2*, *BRIP1*, *CCND1*, *CDH1*, *CDK4*, *CHEK2*, *CYP1B1*, *EGFR*, *EPCAM*, *ERBB2*, *ERBB4*, *ERCC1*, *FANCD2*, *FANCI*, *MLH1*, *MRE11A*, *MSH2*, *MSH6*, *MUTYH*, *NBN*, *NOTCH1*, *PALB2*, *PIK3CA*, *PMS2*, *PTEN*, *RAD50*, *RAD51C*, *RAD51D*, *RET*, *SMAD4*, *STK11*, *TP53*, *XPC*, and *XRCC1* (**Table 1**). Most of the 43 genes, such as *APC*, *ATM*, *ATR*, *BAP1*, *BARD1*, *BLM*, *BRCA1*, *BRCA2*, *BRIP1*, *CDH1*, *CHEK2*, *CYP1B1*, *EPCAM*, *ERCC1*, *FANCD2*, *FANCI*, *MLH1*, *MRE11A*, *MSH2*, *MSH6*, *MUTYH*, *NBN*, *PALB2*, *PMS2*, *PTEN*, *RAD50*, *RAD51C*, *RAD51D*, *STK11*, *XPC*, and *XRCC1* were DDR genes. The panel was composed of the whole coding sequence and splicing region (exonic boundaries ± 10 bp) of each gene. The target region size of the panel was 114 kb, and 99% of the target region was covered with 1,352 amplicons (Analyses, Beijing, China).

**Table 1:** Gene list

	Gene	Transcript	Exon number	Target region bases (bp)
1	*AKT1*	NM_005163	14	3,008
2	*APC*	NM_000038	15	8,683
3	*ATM*	NM_000051	62	9,792
4	*ATR*	NM_001184	47	8,158
5	*BAP1*	NM_004656	17	2,361
6	*BARD1*	NM_000465	11	2,445
7	*BLM*	NM_000057	21	4,465
8	*BRAF*	NM_004333	18	6,459
9	*BRCA1*	NM_007294	22	5,813
10	*BRCA2*	NM_000059	26	10,518
11	*BRIP1*	NM_032043	19	3,941
12	*CCND1*	NM_053056	5	4,238
13	*CDH1*	NM_004360	16	2,810
14	*CDK4*	NM_000075	8	1,865
15	*CHEK2*	NM_001005735	15	1,912
16	*CYP1B1*	NM_000104	2	1,653
17	*EGFR*	NM_005228	28	9,905
18	*EPCAM*	NM_002354	9	1,036
19	*ERBB2*	NM_004448	27	4,557
20	*ERBB4*	NM_005235	28	12,097
21	*ERCC1*	NM_001983	10	3,379
22	*FANCD2*	NM_001018115	43	4,922
23	*FANCI*	NM_018193	36	4,168
24	*MLH1*	NM_000249	19	2,462
25	*MRE11A*	NM_005591	19	2,318
26	*MSH2*	NM_000251	16	2,966
27	*MSH6*	NM_000179	10	4,184
28	*MUTYH*	NM_012222	16	1,861
29	*NBN*	NM_002485	16	2,426
30	*NOTCH1*	NM_017617	34	9,568
31	*PALB2*	NM_024675	13	3,692
32	*PIK3CA*	NM_006218	21	9,259
33	*PMS2*	NM_000535	15	2,740
34	*PTEN*	NM_000314	9	1,303
35	*RAD50*	NM_005732	25	4,190
36	*RAD51C*	NM_058216	9	1,226
37	*RAD51D*	NM_002878	10	1,088
38	*RET*	NM_020975	20	3,578
39	*SMAD4*	NM_005359	11	1,770
40	*STK11*	NM_000455	9	1,393
41	*TP53*	NM_000546	10	1,283
42	*XPC*	NM_004628	16	3,650
43	*XRCC1*	NM_006297	17	2,052

### NGS and data processing

Genomic DNA was extracted from peripheral blood samples (2–5 mL) using a QIAamp DNA Blood Mini Kit (Qiagen, Hilden, Germany) according to the manufacturer’s instructions. The DNA library was constructed using GO prep kits (Analyses), in which every library from different samples was marked with varied indices. The prepared libraries were sequenced by Ion S5 (TMO, Shanghai, China). Qualified reads were aligned to the human reference genome hg19 by TMAP (v.5.10). The target regions were sequenced at a depth > 200 times. Germline mutations (SNV/small InDel < 22 bp) were detected using TVC software (v.5.10). Then, Ensembl Variant Effect Predictor software (http://grch37.ensembl.org/info/docs/tools/vep/index.html) was used for variant interpretation, such as HGVS notation, Population Allele Frequencies from GnomAD (http://gnomad.broadinstitute.org/), 1K Genomes Project (http://www.1000genomes.org), Clinical Significance States assigned by HGMD (http://www.hgmd.cf.ac.uk/), ClinVar (http://www.ncbi.nlm.nih.gov/clinvar/), BRCA Exchange (https://brcaexchange.org/), and BIC (https://research.nhgri.nih.gov/bic/). In addition, all significant mutations, including pathogenic variants, likely pathogenic variants, and variants of uncertain significance (VUS) were confirmed by Sanger sequencing.

### Variant classification

All mutations were classified according to the American College of Medical Genetics (ACMG) professional practice and guidelines^21^. Following the principles of the ACMG, all germline mutations were classified as pathogenic (P), likely pathogenic (LP), uncertain significance (US), likely benign (LB), or benign (B). Each pathogenic criterion was weighted as very strong (PVS1: nonsense, frameshift, canonical ± 1 or 2 splice sites, initiation codon), strong (PS1–4); moderate (PM1–6), or supporting (PP1–5), and each benign criterion was weighted as stand-alone (BA1), strong (BS1–4), or supporting (BP1–6). The other mutations were classified as variants of uncertain significance (VUS).

### Statistical analysis

The correlations between genetic variants and clinicopathological characteristics of patients were determined using the Student’s *t*-test, the chi-square test, or Fisher’s precise test. *P*-values less than 0.05 were considered to be statistically significant.

## Results

### Quality assessment of sequencing data

**Figure 1:**
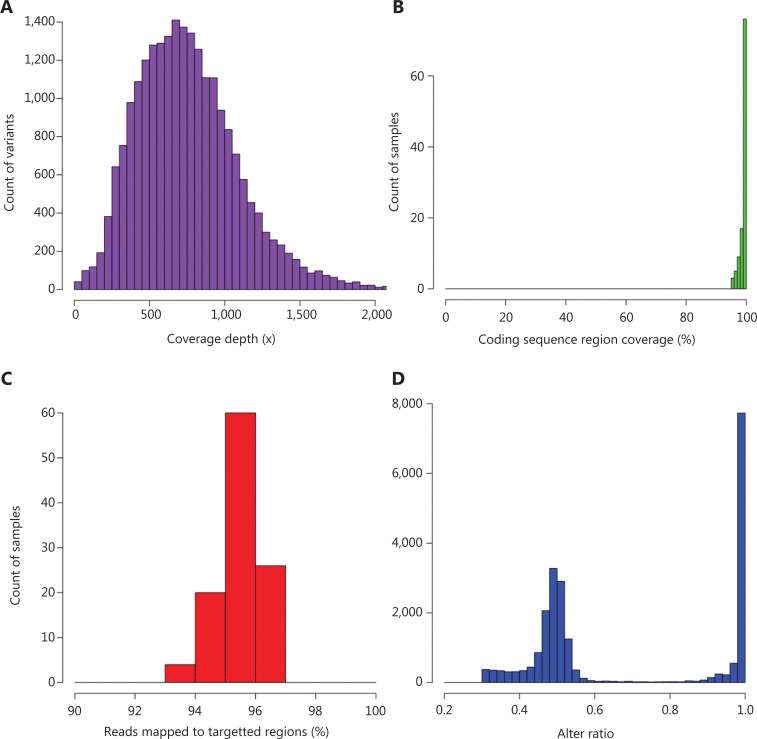
Quality assessment of the sequencing data. (A) The sequencing depth of variants. (B) Percentage of all mapped reads for samples. (C) Percentage of reads mapped to target regions for samples. (D) The distribution of allele fractions across all identified variants.

More than 26.7 GB of sequencing data were generated from 116 clinical samples. An average of 1.0 million reads for each sample was obtained. The depth of each variant was mainly 200–1,500×, and the average depth was more than 800× (**Figure 1A**). On average, 98.9% of all reads could be mapped back to the hg19 genome (**Figure 1B**) and 96.0% of all reads were mapped to targeted regions (**Figure 1C**). After variant calling, the allele fraction plots of all the variants demonstrated a clear bimodal distribution pattern peaking at 0.5 and 1.0, which indicated that a typical distribution pattern of germline mutations was achieved (**Figure 1D**).

### Identification of germline mutations

**Figure 2:**
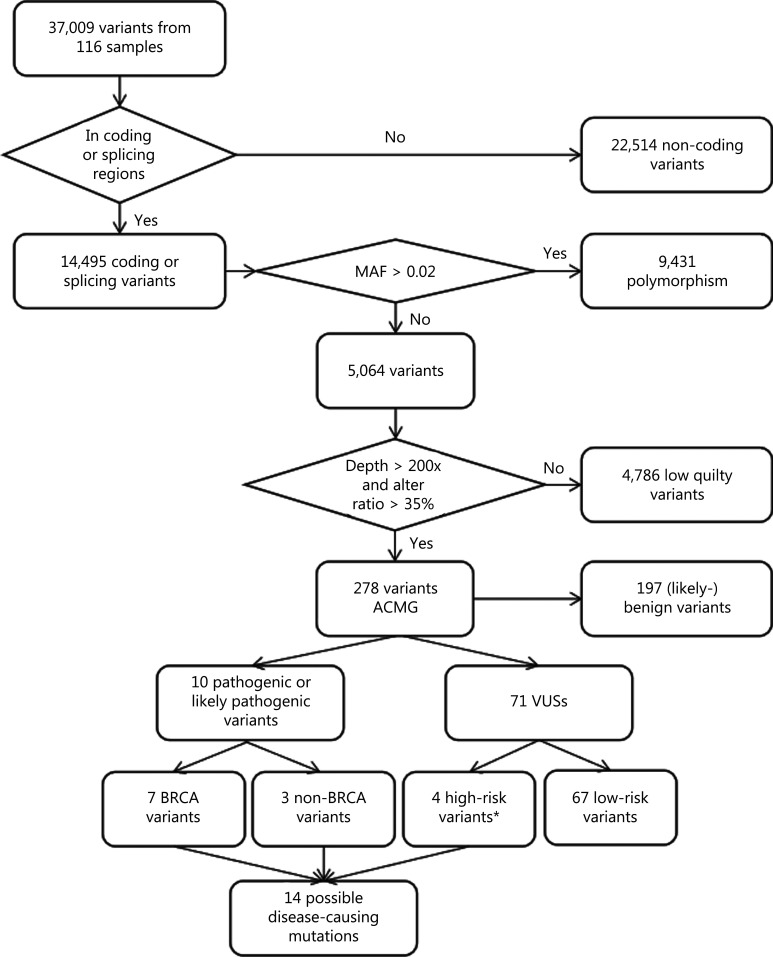
Filtering steps of mutations. *Predicted as damaging by multiple software programs or reported in cancer patients.

**Figure 3:**
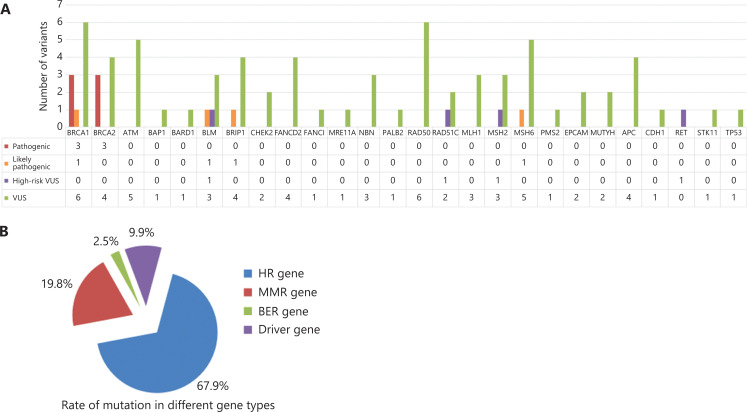
Distribution of the mutated genes. (A) Number of mutations in the mutated genes. (B) Percentage of mutations in different gene types. HR: homologous recombination repair, MMR: mismatch repair, BER: base excision repair.

We detected 37,009 variants among 43 genes in 116 subjects from 27 families. After variant filtering (**Figure 2**), 81 germline mutations in 26 genes were identified in 96 subjects (**Figure 3A**; more details are available in Supporting Information **Supplementary Table S2**). The genes with ≥ 5 mutations were *BRCA1*, *BRCA2*, *ATM*, *BLM*, *BRIP1*, *MSH6*, and *RAD50*. Of the mutated genes, 80.8% (21/26) were DDR genes (*ATM*, *BRCA1*, *BRCA2*, *BAP1*, *BARD1*, *BRIP1*, *BLM*, *CHEK2*, *FANCD2*, *FANCI*, *MRE11A*, *NBN*, *PALB2*, *RAD50*, *RAD51C*, *MLH1*, *MSH2*, *MSH6*, *EPCAM*, *PMS2,* and *MUTYH*), and 19.2% (5/26) were driver genes (*APC*, *CDH1*, *RET*, *STK11*, and *TP53*). Among these DDR genes, 71.4% (15/21) were involved in HR, 23.8% (5/21) were involved in MMR, and 4.8% (1/21) were involved in BER. Of the 81 mutations, 67.9% were found in HR genes, 19.8% in MMR genes, 2.5% in BER genes, and 9.9% in driver genes (**Figure 3B**). More than 90% of the mutations occurred in DDR genes. Of these mutations, 10 (12.3%) were pathogenic or likely pathogenic (P/LP), and 71 (88.7%) were VUS. There were 7 P/LP mutations detected in *BRCA1/2* genes and 3 P/LP mutations detected in *non-BRCA* genes. Four VUS were considered high risk based on software predictions and literature reports. In this article, P/LP mutations and high risk VUS were defined as possible disease-causing mutations^22^.

### The correlation between genetic mutations and hereditary BC families

Among the 27 familial BC families, 48.1% (13/27) had possible disease-causing mutations in known BC predisposition genes, including *BRCA1*, *BRCA2*, *BLM*, *BRIP1*, *MSH2*, *MSH6*, *RAD51C*, and *RET*. The cause of hereditary BC in 51.9% (14/27) of the families was unknown. Hereditary BC in 25.9% (7/27) of the families was associated with *BRCA1/2* genes, while that in 22.2% (6/27) was associated with *non-BRCA* genes (**Table 2**). This showed that testing the *non-BRCA* genes increased the detection of hereditary BC by 22.2%.

**Table 2:** The correlation between mutation genes and cancer types of hereditary breast cancer families

Types of cancer within a family	No. of families	*BRCA*-related families	*Non-BRCA*-related families	Families of unknown reason
**Breast cancer**	11	3 (27.3%)	2 (18.2%)	6 (54.5%)
**Breast cancer + ovarian cancer**	4	2 (50.0%)	0 (0.0%)	2 (50.0%)
**Breast cancer (+ ovarian cancer) other cancers**	12	2 (16.7%)	4 (33.3%)	6 (50.0%)
**Total**	27	7 (25.9%)	6 (22.2%)	14 (51.9%)

Of these 27 families, 11 (40.7%) were characterized by BC only, 4 (14.8%) by both BC and ovarian cancer, and 12 (44.5%) by other cancer types besides BC (and ovarian cancer), such as lung cancer, stomach cancer, esophageal cancer, colorectal cancer, and endometrial cancer (**Table 3**). Of the families with BC only, 27.3% (3/11) were related with *BRCA* genes and 18.2% (2/11) were related with *non-BRCA* genes. However, of the families with cancer types other than BC (and ovarian cancer), more were related with *non-BRCA* genes than *BRCA* genes (33.3% *vs.* 16.7%) (**Table 2**).

**Table 3:** The corresponding variants and the cancer types of each family

Family	The possible corresponding variants	Cancer types of a family
F01	*BRCA2* p.Glu97Term	Breast cancer
F02	*BLM* p.Leu60Ile	Breast cancer
F03	Uncertain	Breast cancer
F04	Uncertain	Breast cancer
F05	*MSH2* p.Met688Ile	Breast cancer
F06	Uncertain	Breast cancer
		Hepatic carcinoma
F07	Uncertain	Breast cancer
F08	Uncertain	Breast cancer
		Colorectal cancer
F09	*BRCA1* p.Glu1836fs	Breast cancer
	*BRIP1* p.Lys222Term	Ovarian cancer
F10	*BRCA2* p.Ser2984Term	Breast cancer
		Esophageal cancer
		Stomach cancer
F11	Uncertain	Breast cancer
		Ovarian cancer
F12	Uncertain	Breast cancer,
		Colorectal cancer
F13	*BRCA1* p.Leu1306fs	Breast cancer
		Ovarian cancer
		Colorectal cancer
		Non-Hodgkin’s
		lymphoma
		Esophageal cancer
F14	Uncertain	Breast cancer
		Ovarian cancer
		Stomach cancer
		Esophageal cancer
		Cervical cancer
		Thyroid cancer
F15	Uncertain	Breast cancer
F16	*RAD51C* p.Arg370Term	Breast cancer
		Colorectal cancer
F17	Uncertain	Breast cancer
F18	*RET* p.Glu632Lys	Breast cancer
		Esophageal cancer
F19	Uncertain	Breast cancer
		Colorectal cancer
		Lung cancer
F20	Uncertain	Breast cancer
		Ovarian cancer
F22	*BRCA1* p.Thr327fs	Breast cancer
F23	*BRCA2* p.Arg2520Term	Breast cancer
F24	Uncertain	Breast cancer
F25	Uncertain	Breast cancer
		Colorectal cancer
		Lung cancer
F27	*BLM* p.Asp1116fs	Breast cancer
		Stomach cancer
		Lung cancer
F28	*BRCA1* p.Asp1362fs	Breast cancer
		Ovarian cancer
F29	*MSH6* p.Arg841fs	Breast cancer
		Endometrial cancer

### The distribution of possible disease-causing mutations in BRCA1/2 genes

Among the mutation carriers, 24.0% (23/96) carried possible disease-causing mutations in *BRCA* genes. Of them, 47.8% (11/23) were carriers of the *BRCA1* gene, and 52.2% (12/23) were carriers of the *BRCA2* gene. Therefore, mutation carriers of the *BRCA2* gene occurred 1.09 times more frequently than those of the *BRCA1* gene in these 27 Chinese hereditary BC families.

**Figure 4:**
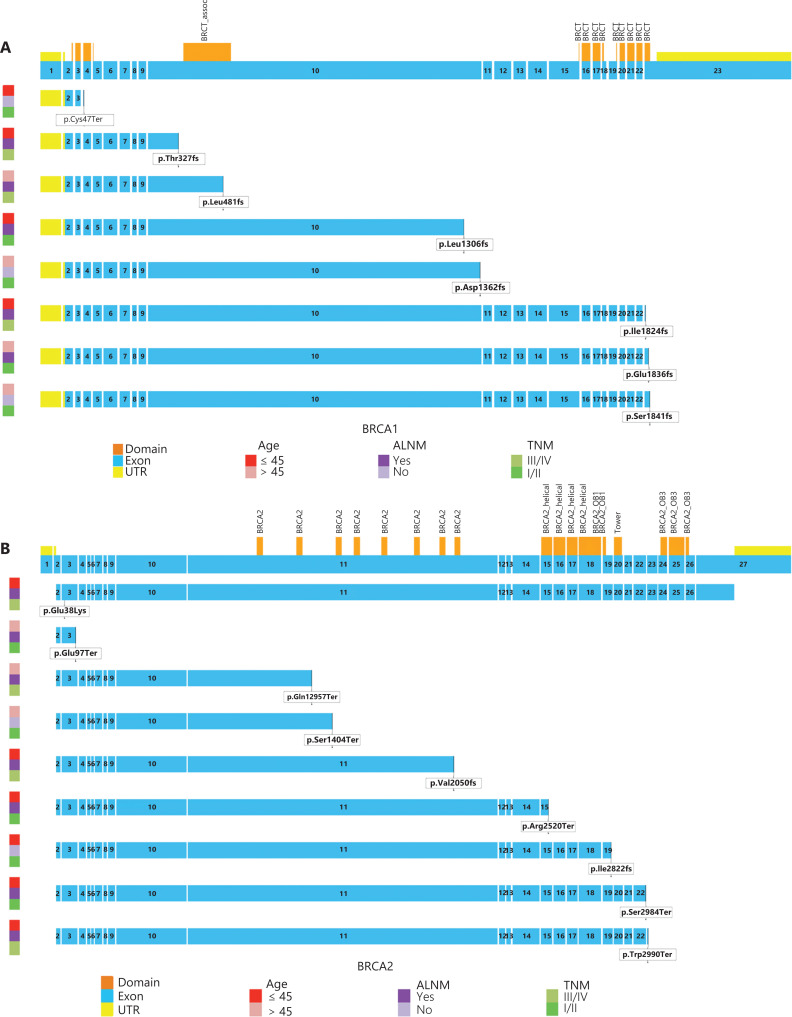
The distribution of *BRCA1/2* mutations in databases (HGMD, ClinVar, and Gnomad) and the disease-causing mutations in *BRCA1/2* genes and its effects on the BRCA protein. (A) The disease-causing mutations in the *BRCA1* gene. (B) The disease-causing mutations in the *BRCA2* gene.

Four possible disease-causing mutations in the *BRCA1* gene were found in 27 familial BC families. There were 3 mutations located in exon 10 and 1 located in exon 23 (**Figure 4A**). *BRCA1* p.Glu1836fs was located in the BRCT2 domain of BRCA1. The BRCT domain is found in a large variety of proteins involved in DNA repair, recombination, and cell cycle control, and functions as a protein-protein interaction module^23,24^. *BRCA1* p.Thr327fs was located upstream of the serine-rich domain associated with BRCT and found in a family with 2 BC patients, 1 of whom was diagnosed with BC at 32 years of age and died of BC. The healthy member with this mutation is taking tamoxifen orally to prevent BC under the guidance of her physician. *BRCA1* p.Asp1362fs and p.Leu1306fs were not located in the functional domain of BRCA1. Both were found in a family with BC and ovarian cancer. Four possible disease-causing mutations in the *BRCA1* gene previously found in 146 sporadic BC patients were located in exons 4, 10, 23, and all in the functional domain^22^. There was no significant difference in the location of these mutations in the *BRCA1* gene between pedigrees and sporadic patients. Of the 8 possible disease-causing mutations in the *BRCA1* gene, 7 (87.5%) were frameshift mutations, and 5 (62.5%) were located in the functional domain of the *BRCA1* gene, especially BRCT2 (**Figure 4A**). The patients with *BRCA1* p.Ile1824fs and p.Leu1306fs were diagnosed with BC at the age of ≤ 45 years and had lymph node metastases. The tumor-node-metastasis (TNM) stage of *BRCA1* p.Ile1824fs mutation carriers was stage III. The patient with *BRCA1* p.Leu481fs was diagnosed with BC at the age of > 45 years but had lymph node metastasis and was in stage III. The *BRCA1* p.Asp1362fs mutation carriers were ≥ 45 years of age, in stage I, and had no lymph node metastasis.

Three possible disease-causing mutations in the *BRCA2* gene were found in 27 familial BC families and located in exons 3, 15, and 23 (**Figure 4B**). *BRCA2* p.Arg2520Term was located in the helical domain of BRCA2. The region interacts with the DSS1 (deleted in split hand/split foot) protein in mammalian cells, which is required for normal cell growth^25^. *BRCA2* p.Glu97Term and p.Ser2984Term were not located in the functional domain of BRCA2. *BRCA2* p.Glu97Term was found in a family with 2 BC patients. The onset age of the patients was over 50 years. *BRCA2* p.Ser2984Term was found in a family with 3 BC patients. Among them, 1 patient developed BC at the age of 35 years, and 1 patient experienced contralateral BC after she was diagnosed with BC at 42 years of age. Six possible disease-causing mutations in the *BRCA2* gene found previously in 146 sporadic BC patients were located in exons 3, 11, 19, and 23. The distribution of possible disease-causing mutations found in sporadic patients may be more dispersed in the *BRCA2* gene^22^. Of the 9 possible disease-causing mutations of the BRCA2 gene, 6 (66.7%) were nonsense mutations, and mutations within exon 11 of *BRCA2* were the most common. We found that most of the mutations carried by patients with an onset age of ≤ 45 years were located in the region of exon 15 or behind exon 15, and 80.0% of the patients with *BRCA2* possible disease-causing mutations had lymph node metastases. The patients with *BRCA2* p.Glu38Lys, p.Val2050fs, p.Arg2520Term, p.Ser2984Term, and p.Trp2990Term were diagnosed with BC at the age of ≤ 45 years and had lymph node metastases. The TNM stage of *BRCA2* p.Glu38Lys, p.Val2050fs, and p.Trp2990Term mutation carriers was stage III. The *BRCA2* p.Ser1404Term mutation carriers were more than 45 years of age, were in stage I, and had no lymph node metastasis.

### Possible disease-causing mutations in non-BRCA genes

Seven possible disease-causing mutations in *non-BRCA* genes were found in 27 familial BC families including 3 likely pathogenic mutations in the *BLM*, *BRIP1*, and *MSH6* genes, and 4 high risk VUSs in the *BLM*, *MSH2*, *RAD50C*, and *RET* genes. *BLM* p.Asp1116fs was not present in population databases such as ExAC, gnomAD, and 1,000 Genomes. This sequence change duplicated 1 nucleotide from exon 17 of the BLM mRNA (c.3349dupA), causing a frameshift variant at codon 1,116. Loss-of-function variants in the *BLM* gene are known to be the pathogenic mechanism for Bloom syndrome^26^. The sequence change of *BRIP1* p.Lys222Term replaced A with T from exon 7 of the BRIP1 mRNA (c.664A>T), causing a nonsense mutation at codon 222. It was also not present in population databases such as ExAC, gnomAD, and 1,000 Genomes and was recorded as pathogenic in the ClinVar database (SCV001187697). *MSH6* p.Arg841fs was not present in population databases such as ExAC, gnomAD, and 1,000 Genomes. This sequence change deleted 1 nucleotide from exon 4 of the MSH6 mRNA (c.2522delG), causing frameshift variants at codon 841. Loss-of-function variants in the *MSH6* gene are known to be the pathogenic mechanism for Lynch syndrome^27,28^. According to the guidelines of the ACMG, these 3 mutations were classified as likely pathogenic.

Four VUS were considered high risk in this study, namely, *BLM* p.Leu60Ile, *MSH2* p.Met688Ile, *RAD50C* p.Arg370Term, and *RET* p.Glu632Lys. *BLM* p.Leu60Ile, *MSH2* p.Met688Ile, and *RET* p.Glu632Lys were predicted to be damaging by multiple software programs. *BLM* p.Leu60Ile was recorded as having conflicting interpretations of pathogenicity in the ClinVar database without clinical information. *MSH2* p.Met688Ile was recorded with uncertain significance in the ClinVar database and reported in colorectal cancer, endometrial cancer, and Lynch syndrome^29–32^. *RET* p.Glu632Lys was recorded with uncertain significance in the ClinVar database and reported in medullary thyroid carcinoma^33,34^, Hirschsprung’s disease^35^, esophageal cancer^36^, colorectal cancer^37^ and sporadic pheochromocytoma^38^. The nonsense mutation *RAD50C* p.Arg370Term exhibited the termination codon at 370 amino acids. It was not clear whether *RAD50C* p.Arg370Term would lead to nonsense mutation-mediated mRNA decay because it was located in the last exon. It was also recorded with uncertain significance in the ClinVar database. Overall, the studies have shown that deletion of the terminal 11 amino acid residues led to cell localization errors of the RAD51C protein^39^.

### Comutation of BRCA and non-BRCA genes in the BC pedigree

**Figure 5:**
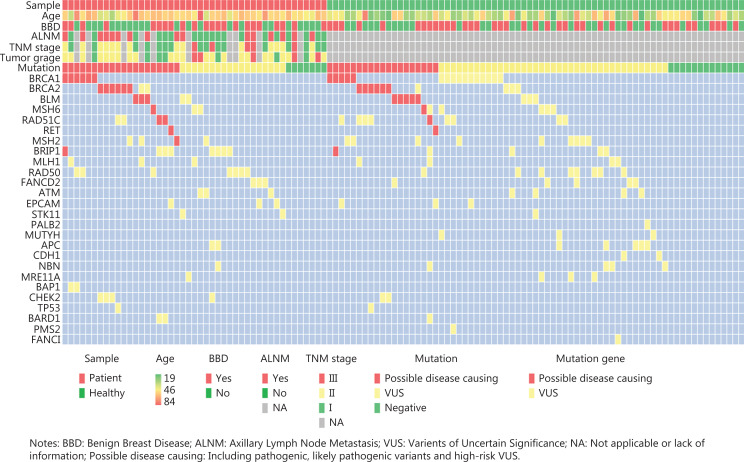
The distribution of mutations and clinical features across all subjects.

In this study, 82.76% (96/116) of all subjects were found to carry at least 1 gene mutation. Only 34.4% (33/96) of the mutation carriers had 1 mutation, while 65.6% (63/96) had ≥ 2 mutations simultaneously. It is common that 1 person carries > 1 mutation, which was detected either in *BRCA1/2* genes or *non-BRCA* genes (**Figure 5**). Twenty-nine (25.0%, 29/116) subjects carried both *BRCA* and *non-BRCA* mutations, namely, 12 patients and 17 healthy members. These non-BRCA mutations occurred in the *ATM*, *BAP1*, *BLM*, *BRIP1*, *CHEK2*, *EPCAM*, *MLH1*, *MSH2*, *MSH6*, *MUTYH*, *PMS2*, *RAD50*, *RAD51C*, and *TP53* genes, 92.9% of which were related to DDR genes. Among the comutation samples of *BRCA* and *non-BRCA* genes, there were 19 carriers with possible disease-causing mutations in *BRCA* genes and 5 carriers with possible disease-causing mutations in *non-BRCA* genes. Two carriers had possible disease-causing mutations in both *BRCA* genes and *non-BRCA* genes. Both were from 1 family with 1 BC patient and 1 ovarian cancer patient.

### The clinicopathological characteristics of hereditary BC patients with varied genetic mutations

According to the mutation status (P, LP, VUS), 36 BC patients were divided into 3 groups: the *BRCA* mutation group (*n* = 9), *non-BRCA* mutation group (*n* = 20), and nonmutation group (*n* = 7) (**Table 4**). The *BRCA* mutation group had a comparably higher risk of axillary lymph node metastasis than the nonmutation group (77.8% *vs.* 28.6%, *P* = 0.049). There was no significant difference in age, unilateral or bilateral, tumor size, TNM stage, tumor grade, histological type, luminal type, history of benign breast diseases, recurrence, or metastasis between the *BRCA* mutation group and the nonmutation group. In contrast, the *non-BRCA* mutation group had a significantly higher occurrence of benign breast disease before BC than the nonmutation group (70.0% *vs.* 14.3%, *P* = 0.021). However, there was no significant difference in other parameters.

**Table 4:** Clinicopathological characteristics among 36 patients with different mutation status

Features	*n*	BRCA+ (*n* = 9, %)	non-BRCA+ (*n* = 20, %)	Negative (*n* = 7, %)	P1-value*	*P2-*value*
**Age of onset (years)**						
Mean ± size		44.8 ± 9.5	55.7 ± 10.3	51.3 ± 6.8	0.148^a^	0.307^a^
≤ 45	8	5 (55.6%)	1 (5.0%)	2 (28.6%)	0.358	0.156
> 45	28	4 (44.4%)	19 (95.0%)	5 (71.4%)		
**Unilateral/bilateral**					0.088	1.000
Unilateral	31	5 (55.6%)	19 (95.0%)	7 (100.0%)		
Bilateral	5	4 (44.4%)	1 (5.0%)	0 (0.0%)		
**Tumor size**					0.550	1.000
≤ 3 cm	28	8 (88.9%)	15 (75.0%)	5 (71.4%)		
> 3 cm	8	1 (11.1%)	5 (25.0%)	2 (28.6%)		
**Axillary lymph node metastasis**					0.049	1.000
Yes	16	7 (77.8%)	7 (35.0%)	2 (28.6%)		
No	20	2 (22.2%)	13 (65.0%)	5 (71.4%)		
**TNM stage**					0.263^b^	0.435^b^
0 + I	17	2 (22.2%)	10 (50.0%)	5 (71.4%)		
II	14	7 (77.8%)	6 (30.0%)	1 (14.3%)		
III	5	0 (0.0%)	4 (20.0%)	1 (14.3%)		
**Tumor grade**					0.109^b^	0.665^b^
I	8	1 (11.1%)	5 (25.0%)	2 (28.6%)		
II	23	8 (88.9%)	12 (60.0%)	3 (42.9%)		
III	5	0 (0.0%)	3 (15.0%)	2 (28.6%)		
**Histological type**					1.000	0.633
Breast invasive ductal carcinoma	28	7 (77.8%)	16 (80.0%)	5 (71.4%)		
Other	8	2 (22.2%)	4 (20.0%)	2 (28.6%)		
**Luminal type**					0.086^b^	0.160^b^
Luminal A	4	0 (0.0%)	3 (15.0%)	2 (28.6%)		
Luminal B	21	6 (66.7%)	12 (60.0%)	5 (71.4%)		
HER2 overexpressing	4	0 (0.0%)	4 (20.0%)	0 (0.0%)		
Triple negative	4	3 (33.3%)	1 (5.0%)	0 (0.0%)		
**With benign breast disease**					0.060	0.024
Yes	21	6 (66.7%)	14 (70.0%)	1 (14.3%)		
No	15	3 (33.3%)	6 (30.0%)	6 (85.7%)		
**Recurrence or metastasis**					1.000	1.000
Yes	2	1 (11.1%)	1 (5.0%)	0 (0.0%)		
No	34	8 (88.9%)	19 (95.0%)	7 (100.0%)		

*Fisher’s precise test; ^a^Student’s *t*-test; ^b^*rank-sum* test; P1-value: *BRCA*+ group *vs*. the negative group; P2-value: *non-BRCA*+ group *vs*. the negative group.

The average onset age of BC in the *BRCA* mutation group was younger than that in the nonmutation group (44.8 ± 9.5 *vs.* 51.3 ± 6.8) (**Table 4**). However, the difference was not statistically significant. The small sample size might be an important factor. We therefore included an additional 47 BC patients with family histories of BC. Among these 83 familial BC patients, 20 had mutations in *BRCA1/2* genes, 25 had *non-BRCA* mutations, and 38 had no mutations. We compared the correlations between *BRCA* mutation status and the clinical and pathological features of patients (**Table 5**). The results showed that the average onset age in the *BRCA* mutation group was significantly younger than that in the nonmutation group (45.7 ± 9.6 *vs.* 51.9 ± 8.7, *P* = 0.015). Furthermore, the percentages of young BC (55.6% *vs.* 28.6%, *P* = 0.023), lymph node metastatic (70.0% *vs.* 28.9%, *P* = 0.005), clinical stage III (35.0% *vs.* 18.4%, *P* = 0.011), and triple-negative BC (40.0% *vs.* 7.9%, *P* = 0.002) were higher in the *BRCA* mutation group than in the nonmutation group. In contrast, no significant difference was detected when comparing the above clinicopathological features between the *non-BRCA* mutation group and the non-mutation group.

**Table 5:** Clinicopathological characteristics among 83 patients with different mutation status

Features	*n*	*BRCA+* (*n* = 20, %)	*non-BRCA*+ (*n* = 25, %)	Negative (*n* = 38, %)	P1-value*	*P2-*value*
**Age of onset**						
Mean ± size		45.7 ± 9.6	53.8 ± 10.7	51.9 ± 8.7	0.015^a^	0.432^a^
≤ 45	23	11 (55.0%)	3 (12.0%)	9 (23.7%)	0.023	0.334
> 45	60	9 (45.0%)	22 (88.0%)	29 (76.3%)		
**Axillary lymph node metastasis**					0.005	1.000
Yes	33	14 (70.0%)	8 (32.0%)	11 (28.9%)		
No	50	6 (30.0%)	17 (68.0%)	27 (71.1%)		
**TNM stage**					0.011^b^	0.273^b^
0 + I	37	4 (20.0%)	10 (40.0%)	23 (60.5%)		
II	27	9 (45.0%)	10 (40.0%)	8 (21.1%)		
III	19	7 (35.0%)	5 (20.0%)	7 (18.4%)		
**Tumor grade**					0.223^b^	0.986^b^
I	14	1 (5.0%)	5 (20.0%)	8 (21.1%)		
II	54	16 (80.0%)	15 (60.0%)	23 (60.5%)		
III	15	3 (15.0%)	5 (20.0%)	7 (18.4%)		
**Luminal type**					0.002^b^	0.293^b^
Luminal A	4	2 (10.0%)	4 (16.0%)	14 (36.8%)		
Luminal B	21	8 (40.0%)	15 (60.0%)	17 (44.7%)		
HER2 overexpressing	11	2 (10.0%)	5 (20.0%)	4 (10.5%)		
Triple negative	12	8 (40.0%)	1 (4.0%)	3 (7.9%)		

*Fisher’s precise test. ^a^Student’s *t*-test; ^b^*rank-sum* test; P1-value: *BRCA*+ group *vs*. the negative group; P2-value: *non-BRCA*+ group *vs*. the negative group.

### Classical pedigree analysis of hereditary BC families

**Figure 6:**
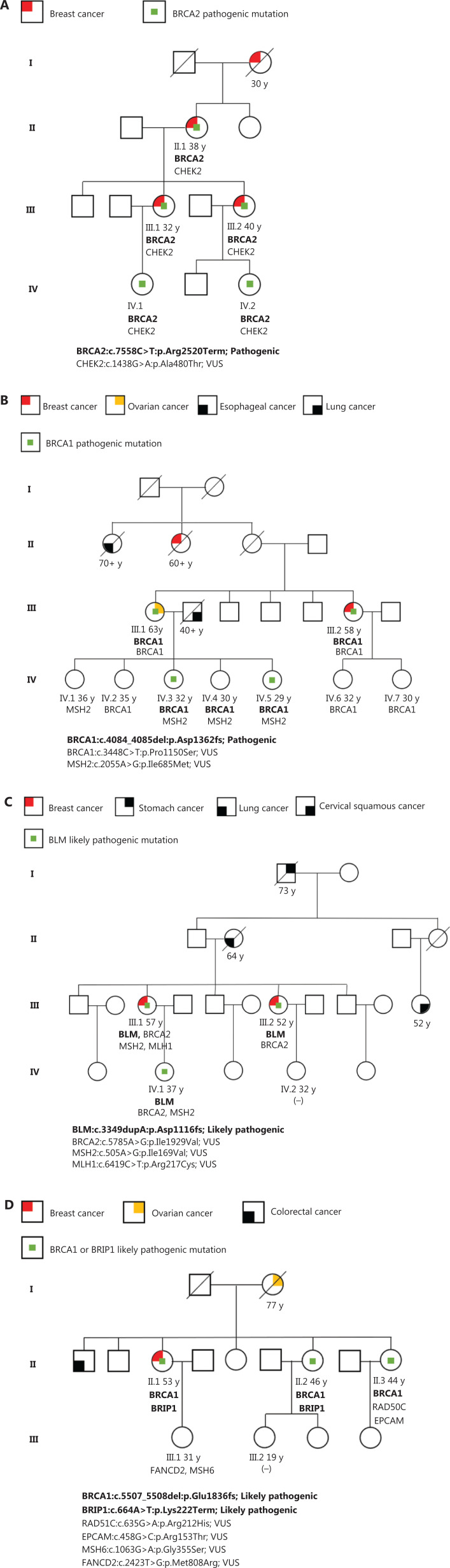
The family tree of classical pedigrees in this study.

**Figure 6** shows several representative families enrolled in this study. As shown in **Figure 6A**, the family included 4 BC patients, of whom 1 died and 1 suffered bilateral BC. We collected samples from other patients. The sequencing results showed that all 3 patients carried *BRCA2* p.Arg2520Term and *CHEK2* p.Ala480Thr mutations. According to the ACMG classification, *CHEK2* p.Ala480Thr is a VUS and *BRCA2* p.Arg2520Term is a pathogenic mutation. Therefore, the *BRCA2* p.Arg2520Term mutation may be the genetic pathogenic mutation of this family. The earliest onset age of BC patients in this family was 30 years of age, and the oldest was 40 years of age. The onset age of BC caused by the *BRCA2* p.Arg2520Term mutation may be earlier. In the fourth generation, 2 young family members, 26 and 22 years of age, respectively, also carried *BRCA2* p.Arg2520Term. Although they are still healthy, it was recommended that prevention and follow-up should be strengthened.

As shown in **Figure 6B**, the pathogenic mutation *BRCA1* Asp1362fs was found in 1 BC patient, 1 ovarian cancer patient, and 2 healthy family members. The BC patient was diagnosed at the age of 58 years, and the ovarian cancer patient was diagnosed at the age of 63 years. BC or ovarian cancer caused by *BRCA1* Asp1362fs may develop later. It is noteworthy that their mother was not a cancer patient, while 1 of their maternal aunts (their mother’s sister) suffered from BC. Genetic testing for these patients was not available because they had died. In addition to BC and ovarian cancer, this family also had 1 esophageal cancer patient. However, there was no definite link between the occurrence of esophageal cancer and the *BRCA1* Asp1362fs mutation. The 2 healthy family members with *BRCA1* Asp1362fs were 32 and 29 years of age, respectively. They may have not reached the age of onset. However, early prevention and regular physical examination were recommended.

**Figure 6C** shows a family with 2 BC patients. Patient III.1 was diagnosed with BC at the age of 57 years. Her sister (Patient III.2) was diagnosed with BC at the age of 52 years. They both carried *BLM* p.Asp1116fs and *BRCA2* p.Ile1929Val. According to the ACMG classification, *BLM* p.Asp1116fs is a likely pathogenic mutation and *BRCA2* p.Ile1929Val is a VUS. The *BLM* gene encodes the DNA helicase RecQ protein on chromosome 15q26, which unwinds a variety of DNA substrates including Holliday junctions, and is involved in several pathways contributing to the maintenance of genome stability^40^. *BLM* p.Asp1116fs was presumably the main genetic cause of BC in this family. One of the healthy family members was also detected with *BLM* p.Asp1116fs at the age of 37 years. Because she might have a high risk of BC, we suggested that she have regular physical examinations for the possible early prevention of BC.

In **Figure 6D**, we collected 5 samples from this family. BC patient II.1 was detected with *BRCA1* p.Glu1836fs and *BRIP1* p.Lys222Term. These 2 mutations are likely pathogenic according to the ACMG guidelines. Her mother had ovarian cancer. Because she had already died, her sample could not be collected. One healthy family member was detected with p.Glu1836fs and *BRIP1* p.Lys222Term and another with *BRCA1* p.Glu1836fs. *BRCA1* p.Glu1836fs has been reported in a Chinese BC patient^41^ and recorded as pathogenic in the LOVD database (https://brcaexchange.org/variant/451386). It was indicated that mutation of BRCA1 p.Glu1836fs can lead to the occurrence of BC without the mutation of *BRIP1* p.Lys222Term. The protein encoded by BRIP1 interacts with the BRCT repeats of BRCA1 protein. The bound complex is important in the normal double-strand break repair function of BRCA1^42^. In this family, healthy carriers of likely pathogenic mutations were also recommended to seek close follow-up and early prevention of BC.

Annual breast magnetic resonance imaging or mammography screening is recommended for younger pathogenic mutation carriers. For healthy women with *BRCA* pathogenic mutations who have no fertility requirements, chemoprevention or risk-reduction surgery is recommended to reduce the risk of BC occurrence^43^.

## Discussion

We performed NGS for 116 subjects from 27 familial BC families based on the Ion Torrent S5 platform. The average sequencing depth was more than 800×. The percentage of hereditary BC in families caused by mutations in known genetic predisposition genes was approximately 48.1%, which was higher than that of other reports of familial BC^44–46^. This was probably because of the high sensitivity of amplicon-based NGS. In addition, the range of genes tested was increased in this study.

In this study, 43 genes were used to detect the hereditary risk of familial BC and other BC-related inherited syndromes. Most of them, such as *AKT1*, *APC*, *ATR*, *ATM*, *BAP1*, *BARD1*, *BLM*, *BRCA1*, *BRCA2*, *BRIP1*, *CDH1*, *CHEK2*, *EPCAM*, *ERBB2*, *ERCC1*, *FANCI*, *MLH1*, *MRE11A*, *MSH2*, *MSH6*, *MUTYH*, *NBN*, *PALB2*, *PIK3CA*, *PMS2*, *PTEN*, *RAD50*, *RAD51C*, *RAD51D*, *RET*, *STK11*, and *TP53* have been reported to be associated with BC susceptibilities^14,47–51^. In addition, there are still some cancer predisposition genes reported in other BC-related inherited syndromes, such as *CYP1B1*, *CCND1*, *CDK4*, *ERBB4*, *FANCD2*, *NOTCH1*, *SMAD4*, *XPC*, and *XRCC1*^52–55^, most of which belong to DDR-related genes.

*BRCA1* and *BRCA2* are 2 well-known high penetration predisposition genes in hereditary BC. Hereditary BC with *BRCA* mutations is more invasive than that without *BRCA* mutations^9,22,56^. In this study, comparative analyses of clinicopathological features also showed that patients with *BRCA* mutations had a younger age of onset, more advanced stage, and higher risk of axillary lymph node metastasis than those without mutations. The mutation prevalence of *BRCA* is distinct in different countries. In this study, *BRCA*-related families accounted for 25.9% of the 27 familial BC families. In a German study including 21,401 families with familial breast or ovarian cancers, the percentage of *BRCA*-related families was 24.0%^44^. According to another study^57^, which analyzed comparative families with ≥ 2 cases of breast and/or ovarian cancer among first- and second-degree relatives, the percentage of *BRCA*-related families was 46.2% in 78 Caucasian families, 68.9% in 29 Ashkenazi Jewish families, and 27.9% in 43 African families. The prevalence of *BRCA1/2* mutations in this study was comparably higher than those reported in other Chinese familial BC cohorts. According to previous studies^6,22,56^, the prevalence of *BRCA1/2* mutations was 12.7%–19.1% in Chinese familial BC patients, which is distinct from the prevalence of *BRCA1/2* mutations in this study. The disparity might be caused by differences in the study populations and the methods of statistical analyses. In our study, we focused on the prevalence of *BRCA1/2* mutations in each hereditary BC family, including both familial BC patients and their direct relatives. The prevalence of *BRCA1/2* mutation was significantly higher compared to those studies including familial BC patients only. In addition, different geographical areas, ethnic groups, genetic testing methods, as well as limited sample sizes might have contributed to the disparity in the prevalence of *BRCA1/2* mutations. We plan to enroll more hereditary BC families in the future to validate our findings.

We found that the mutation frequency of the *BRCA2* gene was 1.09 times that of the *BRCA1* gene in these BC families. In other reports based on a Chinese population, Zhang et al.^46^ reported in 409 Chinese familial BC patients that the *BRCA2* mutation frequency was 1.7 times (6.6%/3.9%) higher than the *BRCA1* mutation frequency. However, this result was inconsistent with other reported findings, which indicated that the mutation frequency of the *BRCA1* gene was considerably higher than that of the *BRCA2* gene in European and American populations^57,58^. Another important difference is the penetrance of *BRCA*. It is well-documented that Western women who carry a pathogenic *BRCA1* or *BRCA2* mutation may have a 57%–65% or 45%–49% risk of developing BC by the age of 70 years^59,60^. Women of Ashkenazi Jewish and Icelandic descent who carry a *BRCA1/2* mutation have a BC risk as high as 70% by the age of 70 years^3,61,62^. However, the breast cancer risk for *BRCA1/2* mutation carriers is only 35%–49% in women from Australia, the UK, and the Republic of Korea^3,63,64^. A study based on a Chinese population^65^ reported that the estimated cumulative risks of BC by the age of 70 years were 37.9% for *BRCA1* mutation carriers and 36.5% for *BRCA2* mutation carriers. The differences might be predominantly derived from the disparities in ethnic groups, which should be thoroughly investigated in a large-scale random case-control trial, especially in the Chinese population.

Of the 116 subjects in this study, 48.6% were found to exclusively carry mutations in *non-BRCA* genes. Testing for *non-BRCA* genes increased the detection of hereditary BC families by 22.2%. Similar results were also found in other reports. According to the Lin et al.^66^ study, the mutation prevalence of *BRCA1/2* in Han Chinese patients with early onset or with a significant family history was 15.0%, and there was a 7.5% mutation of *non-BRCA* genes in women who tested negative for *BRCA1/2* mutations. In another study of German BC patients, extended testing beyond *BRCA1/2* also identified a deleterious mutation in an additional 6% of patients^67^. Our previous findings also showed that the percentage of possible disease-causing mutation carriers among BC patients with a family history increased from 21.3% to 27.7% when the sequenced genes were increased from 6 to 20^22^. Therefore, broader panel testing including more genes would significantly increase the detection percentage of mutation carriers and enhance the screening efficiency for hereditary BC.

We also found that *non-BRCA*-mutated BC was more likely to be accompanied by benign breast diseases. Benign breast disease is an important risk factor for the development of BC. It has been reported that women with severe atypical epithelial hyperplasia of the breasts were twice as likely to develop BC as women without such diseases^68^. The *non-BRCA* gene mutation has a weaker pathogenic effect on carcinogenesis than the *BRCA* gene^69,70^. It is reasonable that BC gradually develops from benign breast disease upon stimulation from *non-BRCA* gene mutations. This could also explain the trend to some degree that the average age of onset of BC patients with *non-BRCA* mutations was older than that of *BRCA*-mutated BC patients (55.84 ± 10.50 *vs.* 45.50 ± 9.24). These findings indicated that genetic variants of those *non-BRCA* genes also played an important role in the development of hereditary BC. These genetic variants should be further evaluated to predict the hereditary BC predisposition of high risk individuals.

In this study, 80.8% (21/26) of the mutated genes were DDR genes. Among these, 71.4% of DDR genes were involved in the HR pathway. In addition to *BRCA1/2* genes, the mutated HR genes also included *ATM*, *BAP1*, *BARD1*, *BRIP1*, *BLM*, *CHEK2*, *FANCD2*, *FANCI*, *MRE11A*, *NBN*, *PALB2*, *RAD50*, and *RAD51C*. The interaction between BRCA1-BARD1, the BRCA2-PALB2 complex, and the recombinant enzyme RAD51 is an important aspect in the HR process^71^. The most important function of HR genes is to repair DNA double strand breaks (DSBs), which are the most serious type of DNA damage^72,73^. Breast cells with homologous recombination repair defects may not be able to initiate HR to repair DSBs. Abnormal repair may lead to chromosome loss, transposition, and other changes. Over time, under the influence of multiple carcinogenic factors, the accumulation of errors leads to the development of BC^74^. The mutated MMR genes included *MLH1*, *MSH2*, *MSH6*, *EPCAM*, and *PMS2*, accounting for 23.8% of the DDR genes. MMR genes prevent mutational events through correction of mismatched bases during DNA replication. Genetic defects in the DNA MMR system result in DNA replication errors, including base substitutions and insertion-deletion loops, known as microsatellite instability^75^. Germline mutations in MMR genes can give rise to Lynch syndrome (LS), an autosomal-dominant cancer predisposition syndrome that increases the risk for several forms of malignancy, including colorectal (lifetime cancer risk, 70%–80%), endometrial (50%–60%), stomach cancer (13%–19%), and ovarian cancer (9%–14%). BC incidence has been found to be increased in patients with Lynch syndrome^76^. MMR genes belong to low penetrance genes associated with BC. Studies have suggested that there might be a functional overlap between the MMR and FA-BRCA pathways^77,78^. Furthermore, 19.2% of the mutated genes were driver genes, including *APC*, *CDH1*, *RET*, *STK11*, and *TP53*. Driver gene mutations promote cancer progression and have major impacts on patient clinical outcomes. Further research on these genes in BC tissue may be warranted. Other studies have shown that the mutation clonality of driver genes was prognostic and predictive for BC patients^79,80^.

## Conclusions

In conclusion, this study primarily compared germline mutation profiling among 27 Chinese familial/hereditary BC families to comprehensively evaluate the genetic variants and clinical significance of 43 BC predisposition genes with different penetration rates in carcinogenesis. We found that in addition to *BRCA1/2*, genetic variants in *non-BRCA* genes, especially DDR genes, played significant roles in the development of Chinese familial/hereditary BC, which implied the indispensable significance of more extensive multiple-gene panel testing in genetic screening of hereditary BC families. People with *non-BRCA* gene mutations are more likely to suffer from BC accompanied by benign breast diseases because *non-BRCA* gene mutations have a weaker pathogenic effect on carcinogenesis than *BRCA* genes. Therefore, more intensive mammary screening of *non-BRCA* mutation-bearing individuals in hereditary BC families is recommended to increase the efficacy of early diagnosis and early treatment of BC. However, this study was limited by a small sample size from a single center. A larger multicenter study in a Chinese population should be conducted to validate the findings of this study.
