# Supplementary material for: The mutation landscape of multiple cancer predisposition genes in Chinese familial/hereditary breast cancer families
Source: Cancer Biol Med. 2021 Sep 28;19(6):850–70. doi: 10.20892/j.issn.2095-3941.2021.0011 (PMC9257317; doi:10.20892/j.issn.2095-3941.2021.0011)
Supplement: Supplementary file 1 [file cbm-19-850-s001.pdf]

Supplementary materials

Table S1 The clinicopathological data and mutation information of 47 familial breast cancer patients

Sample ID	Age	Gender	Axillary lymph node metastasis	TNM Stage	Luminal Type	Histological Grade	Mutation
B00010	40	Female	Yes	III	4	II	*BRCA1* p.Ile1824fs
B00011	40	Female	Yes	III	4	II	*BRCA1* p.Ile1824fs
B00014	45	Female	Yes	III	4	II	
B00015	31	Female	No	II	2	II	*PTEN* p.352_352del
B00018	51	Female	No	I	1	I	
B00057	56	Female	No	II	2	III	*PALB2* p.Leu253fs
B00064	40	Female	Yes	III	2	II	
B00068	51	Female	No	I	1	I	
B00071	61	Female	No	II	2	III	
B00072	26	Female	Yes	III	4	II	*BRCA2* p.Val2050fs
B00073	41	Female	No	I	3	III	
B00081	46	Female	Yes	III	3	III	*CHEK2* p.Arg180Cys
B00084	44	Female	No	I	2	I	
B00086	50	Female	No	I	1	II	
B00091	59	Female	No	I	1	II	
B00100	49	Female	No	I	1	II	
B00107	42	Female	No	I	2	II	
B00110	44	Female	No	I	4	III	*BRCA1* p.Cys47Term
B00112	53	Female	No	I	3	III	
B00116	48	Female	Yes	III	2	II	
B00122	55	Female	No	I	2	I	
B00125	52	Female	No	II	3	II	*BRCA1* p.Glu1527Met
B00127	51	Female	Yes	III	1	II	*BRCA2* p.Gln1295Term
B00130	76	Female	Yes	II	1	II	
B00135	49	Female	No	II	1	II	
B00138	44	Female	No	II	1	II	*PMS2* p.Arg169His
B00139	58	Female	Yes	II	3	II	
B00140	71	Female	No	I	1	I	
B00141	59	Female	No	I	2	II	
B00244	59	Female	No	II	3	III	*BRCA1* p.Ser1841fs
B00257	34	Female	No	II	2	II	
B00260	62	Female	No	I	4	II	
B00264	54	Female	No	I	1	II	

Table S1 Continued

Sample ID	Age	Gender	Axillary lymph node metastasis	TNM Stage	Luminal Type	Histological Grade	Mutation
B00266	56	Female	No	II	2	II	*CHEK2* p.Gln439Term; *BARD1* p.359_365del
B00269	60	Female	Yes	III	2	III	
B00278	59	Female	Yes	III	1	II	*BRCA1* p.Leu481fs
B00285	59	Female	No	II	1	I	
B00289	47	Female	No	I	2	III	
B00290	53	Female	Yes	II	1	II	
B00291	49	Female	No	I	1	II	
B00292	52	Female	No	I	2	II	
B00293	57	Female	No	I	3	II	
B00294	47	Female	Yes	III	4	II	
B00296	40	Female	Yes	III	2	III	*BRCA2* p.Trp2990Term
B00297	56	Female	Yes	III	4	II	*BRCA1* p.Ser1363Leu
B00316	43	Female	No	I	2	II	*BRCA2* p.Ile2822fs
B00328	37	Female	Yes	III	2	II	

Note: Luminal Type: 1 = Luminal A; 2 = Luminal B; 3 = HER2 overexpressing; 4 = Triple negative

Table S2 List of mutations

Family ID	Sample ID	Gene	NM	Exon	Genome	AA_change
F01	B00162_190213	*MSH2*	NM_000251	exon1	c.23C>T	p.Thr8Met
F01	B00162_190213	*BRCA2*	NM_000059	exon3	c.289G>T	p.Glu97Term
F01	B00162_190213	*BRCA2*	NM_000059	exon11	c.2519T>C	p.Met840Thr
F01	B00583_190213	*BRCA2*	NM_000059	exon3	c.289G>T	p.Glu97Term
F01	B00584_190213	*RAD50*	NM_005732	exon8	c.1211A>G	p.Gln404Arg
F01	B00584_190213	*MSH2*	NM_000251	exon1	c.23C>T	p.Thr8Met
F01	B00584_190213	*MRE11A*	NM_005591	exon6	c.469A>G	p.Met157Val
F01	B00585_190213	*RAD50*	NM_005732	exon8	c.1211A>G	p.Gln404Arg
F01	B00585_190213	*MSH6*	NM_000179	exon10	c.4065_4066insTTGA	p.Thr1355fs
F01	B00585_190213	*MSH2*	NM_000251	exon1	c.23C>T	p.Thr8Met
F01	B00585_190213	*MRE11A*	NM_005591	exon6	c.469A>G	p.Met157Val
F02	B00662	*BLM*	NM_000057	exon3	c.178T>A	p.Leu60Ile
F02	B00663	*BLM*	NM_000057	exon3	c.178T>A	p.Leu60Ile
F02	B00663	*FANCD2*	NM_001018115	exon29	c.2723C>T	p.Thr908Ile
F02	B00664	*BLM*	NM_000057	exon3	c.178T>A	p.Leu60Ile
F02	B00665	*BLM*	NM_000057	exon3	c.178T>A	p.Leu60Ile
F02	B00666	*BLM*	NM_000057	exon3	c.178T>A	p.Leu60Ile

Table S2 Continued

Family ID	Sample ID	Gene	NM	Exon	Genome	AA_change
F03	B00672	*APC*	NM_000038	exon16	c.5363G>A	p.Arg1788His
F03	B00672	*CHEK2*	NM_001005735	exon7	c.884G>A	p.Ser295Asn
F03	B00672	*BRIP1*	NM_032043	exon5	c.430G>A	p.Ala144Thr
F03	B00674	*APC*	NM_000038	exon16	c.5363G>A	p.Arg1788His
F03	B00674	*NBN*	NM_002485	exon11	c.1690G>A	p.Glu564Lys
F03	B00674	*BRIP1*	NM_032043	exon5	c.430G>A	p.Ala144Thr
F03	B00675	*FANCD2*	NM_001018115	exon6	c.416T>C	p.Leu139Pro
F03	B00675	*APC*	NM_000038	exon16	c.5363G>A	p.Arg1788His
F03	B00676	*BRIP1*	NM_032043	exon5	c.430G>A	p.Ala144Thr
F03	B00676	*APC*	NM_000038	exon16	c.5363G>A	p.Arg1788His
F03	B00676	*NBN*	NM_002485	exon11	c.1690G>A	p.Glu564Lys
F03	B00679	*FANCD2*	NM_001018115	exon6	c.416T>C	p.Leu139Pro
F04	B00557_190213	*EPCAM*	NM_002354	exon4	c.458G>C	p.Arg153Thr
F04	B00557_190213	*BRCA1*	NM_007294	exon23	c.5566C>A	p.Pro1856Thr
F04	B00558_190213	*FANCD2*	NM_001018115	exon34	c.3446C>T	p.Ala1149Val
F04	B00558_190213	*EPCAM*	NM_002354	exon4	c.458G>C	p.Arg153Thr
F05	B00563_190228	*MSH2*	NM_000251	exon13	c.2064G>A	p.Met688Ile
F06	B00571	*STK11*	NM_000455	exon5	c.608C>T	p.Pro203Leu
F06	B00571	*BLM*	NM_000057	exon15	c.2839A>G	p.Ile947Val
F06	B00572	*STK11*	NM_000455	exon5	c.608C>T	p.Pro203Leu
F06	B00574	*STK11*	NM_000455	exon5	c.608C>T	p.Pro203Leu
F06	B00574	*BLM*	NM_000057	exon15	c.2839A>G	p.Ile947Val
F06	B00574	*ATM*	NM_000051	exon3	c.125A>G	p.His42Arg
F06	B00579_190131	*BLM*	NM_000057	exon15	c.2839A>G	p.Ile947Val
F07	B00592	*RAD50*	NM_005732		IVS15+4A>T	
F07	B00593	*RAD50*	NM_005732		IVS15+4A>T	
F07	B00593	*BRIP1*	NM_032043	exon4	c.370A>G	p.Thr124Ala
F07	B00594	*RAD50*	NM_005732		IVS15+4A>T	
F07	B00594	*BRIP1*	NM_032043	exon4	c.370A>G	p.Thr124Ala
F07	B00595	*RAD50*	NM_005732		IVS15+4A>T	
F07	B00595	*BRCA2*	NM_000059	exon10	c.1741T>C	p.Ser581Pro
F09	B00645	*BRCA1*	NM_007294	exon23	c.5507_5508del	p.Glu1836fs
F09	B00645	*BRIP1*	NM_032043	exon7	c.664A>T	p.Lys222Term
F09	B00646	*BRIP1*	NM_032043	exon7	c.664A>T	p.Lys222Term
F09	B00646	*BRCA1*	NM_007294	exon23	c.5507_5508del	p.Glu1836fs

Table S2 Continued

Family ID	Sample ID	Gene	NM	Exon	Genome	AA_change
F09	B00647	*RAD51C*	NM_058216	exon4	c.635G>A	p.Arg212His
F09	B00647	*EPCAM*	NM_002354	exon4	c.458G>C	p.Arg153Thr
F09	B00647	*BRCA1*	NM_007294	exon23	c.5507_5508del	p.Glu1836fs
F09	B00649	*FANCD2*	NM_001018115	exon26	c.2423T>G	p.Met808Arg
F09	B00649	*MSH6*	NM_000179	exon4	c.1063G>A	p.Gly355Ser
F10	B00627_190131	*RAD51C*	NM_058216	exon4	c.635G>A	p.Arg212His
F10	B00627_190131	*BRCA2*	NM_000059	exon22	c.8951C>G	p.Ser2984Term
F10	B00628_190131	*TP53*	NM_000546	exon3	c.91G>A	p.Val31Ile
F10	B00628_190131	*RAD51C*	NM_058216	exon4	c.635G>A	p.Arg212His
F10	B00628_190131	*BRCA2*	NM_000059	exon22	c.8951C>G	p.Ser2984Term
F10	B00629_190131	*RAD51C*	NM_058216	exon4	c.635G>A	p.Arg212His
F10	B00629_190131	*MLH1*	NM_000249	exon9	c.704A>T	p.Asp235Val
F10	B00629_190131	*BRCA2*	NM_000059	exon22	c.8951C>G	p.Ser2984Term
F10	B00630_190131	*RAD51C*	NM_058216	exon4	c.635G>A	p.Arg212His
F10	B00630_190131	*BRCA2*	NM_000059	exon22	c.8951C>G	p.Ser2984Term
F10	B00631_190131	*NBN*	NM_002485	exon11	c.1809C>A	p.Phe603Leu
F10	B00631_190131	*MLH1*	NM_000249	exon9	c.704A>T	p.Asp235Val
F10	B00632_190131	*MLH1*	NM_000249	exon9	c.704A>T	p.Asp235Val
F10	B00632_190131	*FANCI*	NM_018193	exon23	c.2366C>T	p.Ala789Val
F10	B00633_190131	*TP53*	NM_000546	exon3	c.91G>A	p.Val31Ile
F10	B00633_190131	*RAD51C*	NM_058216	exon4	c.635G>A	p.Arg212His
F10	B00633_190131	*BRCA2*	NM_000059	exon22	c.8951C>G	p.Ser2984Term
F10	B00910	*RAD51C*	NM_058216	exon4	c.635G>A	p.Arg212His
F10	B00911	*BRCA1*	NM_007294	exon10	c.743delinsG	p.Thr248Ser
F10	B00911	*RAD51C*	NM_058216	exon4	c.635G>A	p.Arg212His
F10	B00911	*PMS2*	NM_000535	exon11	c.1154_1156del	p.Ile385del
F10	B00912	*RAD51C*	NM_058216	exon4	c.635G>A	p.Arg212His
F10	B00912	*APC*	NM_000038	exon16	c.5081G>T	p.Gly1694Val
F11	B00600	*ATM*	NM_000051	exon57	c.8393C>A	p.Ala2798Asp
F11	B00601	*ATM*	NM_000051	exon57	c.8393C>A	p.Ala2798Asp
F11	B00601	*APC*	NM_000038	exon16	c.8332G>T	p.Ala2778Ser
F11	B00602	*BLM*	NM_000057	exon5	c.1044G>A	p.Met348Ile
F11	B00607	*FANCD2*	NM_001018115	exon34	c.3446C>T	p.Ala1149Val
F12	B00719	*PALB2*	NM_024675	exon4	c.1492G>T	p.Asp498Tyr
F12	B00719	*APC*	NM_000038	exon16	c.8291C>G	p.Ser2764Cys

Table S2 Continued

Family ID	Sample ID	Gene	NM	Exon	Genome	AA_change
F13	B00656	*BRCA1*	NM_007294	exon10	c.3916_3917del	p.Leu1306fs
F13	B00656	*MLH1*	NM_000249	exon8	c.649C>T	p.Arg217Cys
F13	B00656	*BAP1*	NM_004656		IVS5-4C>T	
F13	B00657	*RAD50*	NM_005732	exon25	c.3836G>A	p.Arg1279His
F13	B00657	*BRCA1*	NM_007294	exon10	c.3916_3917del	p.Leu1306fs
F13	B00657	*BAP1*	NM_004656		IVS5-4C>T	
F13	B00658	*BRCA1*	NM_007294	exon10	c.3916_3917del	p.Leu1306fs
F13	B00658	*RAD50*	NM_005732	exon25	c.3836G>A	p.Arg1279His
F14	B00688	*EPCAM*	NM_002354	exon4	c.458G>C	p.Arg153Thr
F14	B00689	*CDH1*	NM_004360	exon12	c.1888C>G	p.Leu630Val
F14	B00689	*ATM*	NM_000051	exon3	c.173A>G	p.Asp58Gly
F14	B00689	*RAD50*	NM_005732	exon3	c.243G>C	p.Gln81His
F15	B00710	*BRCA2*	NM_000059	exon11	c.5785A>G	p.Ile1929Val
F15	B00710	*ATM*	NM_000051	exon14	c.2149C>T	p.Arg717Trp
F15	B00710	*BRIP1*	NM_032043	exon17	c.2440C>T	p.Arg814Cys
F15	B00711	*BRCA1*	NM_007294	exon10	c.3344_3346del	p.1115_1116del
F15	B00712	*BRCA2*	NM_000059	exon18	c.8187G>T	p.Lys2729Asn
F15	B00713	*BRIP1*	NM_032043	exon17	c.2440C>T	p.Arg814Cys
F15	B00714	*ATM*	NM_000051	exon14	c.2149C>T	p.Arg717Trp
F15	B00714	*MSH6*	NM_000179	exon10	c.4065_4066insTTGA	p.Thr1355fs
F16	B00725	*RAD51C*	NM_058216	exon9	c.1108C>T	p.Arg370Term
F16	B00725	*BRIP1*	NM_032043	exon7	c.797C>T	p.Thr266Met
F16	B00728	*BRIP1*	NM_032043	exon7	c.797C>T	p.Thr266Met
F16	B00728	*RAD51C*	NM_058216	exon9	c.1108C>T	p.Arg370Term
F16	B00728	*MLH1*	NM_000249	exon18	c.2101C>A	p.Gln701Lys
F16	B00728	*NBN*	NM_002485	exon11	c.1809C>A	p.Phe603Leu
F16	B00728	*MSH6*	NM_000179	exon10	c.4065_4066insTTGA	p.Thr1355fs
F16	B00728	*BARD1*	NM_000465	exon10	c.1972C>T	p.Arg658Cys
F16	B00729	*MUTYH*	NM_012222	exon12	c.1109C>T	p.Ala370Val
F16	B00730	*BRIP1*	NM_032043	exon7	c.797C>T	p.Thr266Met
F16	B00730	*RAD51C*	NM_058216	exon9	c.1108C>T	p.Arg370Term
F16	B00730	*BARD1*	NM_000465	exon10	c.1972C>T	p.Arg658Cys
F16	B00731	*BRCA1*	NM_007294		IVS2-5->G	
F16	B00731	*MSH6*	NM_000179	exon6	c.3488A>T	p.Glu1163Val
F16	B00731	*MUTYH*	NM_012222	exon12	c.1109C>T	p.Ala370Val

Table S2 Continued

Family ID	Sample ID	Gene	NM	Exon	Genome	AA_change
F17	B00733	*CDH1*	NM_004360	exon12	c.1888C>G	p.Leu630Val
F17	B00734	*MSH6*	NM_000179	exon1	c.88G>A	p.Glu30Lys
F18	B00745	*RET*	NM_020975	exon11	c.1894G>A	p.Glu632Lys
F18	B00745	*EPCAM*	NM_002354	exon3	c.233T>G	p.Leu78Arg
F18	B00746	*RET*	NM_020975	exon11	c.1894G>A	p.Glu632Lys
F18	B00746	*EPCAM*	NM_002354	exon3	c.233T>G	p.Leu78Arg
F18	B00746	*BRIP1*	NM_032043	exon5	c.430G>A	p.Ala144Thr
F19	B00739	*MSH2*	NM_000251	exon3	c.505A>G	p.Ile169Val
F19	B00739	*RAD50*	NM_005732	exon3	c.323A>G	p.Lys108Arg
F19	B00739	*FANCD2*	NM_001018115	exon34	c.3446C>T	p.Ala1149Val
F19	B00741	*FANCD2*	NM_001018115	exon34	c.3446C>T	p.Ala1149Val
F19	B00742	*MSH2*	NM_000251	exon3	c.505A>G	p.Ile169Val
F20	B00619_190228	*MSH6*	NM_000179	exon4	c.2028A>T	p.Lys676Asn
F20	B00619_190228	*MLH1*	NM_000249	exon18	c.2101C>A	p.Gln701Lys
F22	B00603	*BRCA1*	NM_007294	exon10	c.981_982del	p.Thr327fs
F22	B00604	*MSH2*	NM_000251	exon1	c.23C>T	p.Thr8Met
F22	B00604	*ATM*	NM_000051	exon50	c.7382G>A	p.Arg2461His
F23	B00789_190228	*BRCA2*	NM_000059	exon15	c.7558C>T	p.Arg2520Term
F23	B00789_190228	*CHEK2*	NM_001005735	exon14	c.1567G>A	p.Ala523Thr
F23	B00790_190228	*BRCA2*	NM_000059	exon15	c.7558C>T	p.Arg2520Term
F23	B00790_190228	*CHEK2*	NM_001005735	exon14	c.1567G>A	p.Ala523Thr
F23	B00791_190228	*BRCA2*	NM_000059	exon15	c.7558C>T	p.Arg2520Term
F23	B00791_190228	*CHEK2*	NM_001005735	exon14	c.1567G>A	p.Ala523Thr
F23	B00792_190228	*BRCA2*	NM_000059	exon15	c.7558C>T	p.Arg2520Term
F23	B00792_190228	*CHEK2*	NM_001005735	exon14	c.1567G>A	p.Ala523Thr
F23	B00793_190228	*CHEK2*	NM_001005735	exon14	c.1567G>A	p.Ala523Thr
F23	B00793_190228	*BRCA2*	NM_000059	exon15	c.7558C>T	p.Arg2520Term
F24	B00803_190313	*BLM*	NM_000057	exon14	c.2744C>T	p.Ala915Val
F24	B00803_190313	*MRE11A*	NM_005591	exon6	c.469A>G	p.Met157Val
F24	B00804_190313	*MRE11A*	NM_005591	exon6	c.469A>G	p.Met157Val
F24	B00804_190313	*RAD50*	NM_005732		IVS15+4A>T	
F24	B00806_190313	*RAD50*	NM_005732		IVS15+4A>T	
F25	B00810_190403	*BRCA2*	NM_000059	exon24	c.9160delC	p.Pro3054fs
F25	B00810_190403	*NBN*	NM_002485	exon5	c.511A>G	p.Ile171Val
F25	B00811_190403	*BRCA1*	NM_007294	exon10	c.4046C>T	p.Thr1349Met

Table S2 Continued

Family ID	Sample ID	Gene	NM	Exon	Genome	AA_change
F25	B00812_190403	*BRCA1*	NM_007294	exon10	c.4046C>T	p.Thr1349Met
F25	B00813_190403	*RAD51C*	NM_058216	exon1	c.34C>T	p.Arg12Trp
F25	B00813_190403	*BRCA1*	NM_007294	exon10	c.4046C>T	p.Thr1349Met
F27	B00835_190425	*BRCA2*	NM_000059	exon11	c.5785A>G	p.Ile1929Val
F27	B00835_190425	*MLH1*	NM_000249	exon8	c.649C>T	p.Arg217Cys
F27	B00835_190425	*MSH2*	NM_000251	exon3	c.505A>G	p.Ile169Val
F27	B00835_190425	*BLM*	NM_000057	exon17	c.3349dupA	p.Asp1116fs
F27	B00836_190425	*BRCA2*	NM_000059	exon11	c.5785A>G	p.Ile1929Val
F27	B00836_190425	*BLM*	NM_000057	exon17	c.3349dupA	p.Asp1116fs
F27	B00839_190425	*MSH2*	NM_000251	exon3	c.505A>G	p.Ile169Val
F27	B00839_190425	*BRCA2*	NM_000059	exon11	c.5785A>G	p.Ile1929Val
F27	B00839_190425	*BLM*	NM_000057	exon17	c.3349dupA	p.Asp1116fs
F28	B00875	*BRCA1*	NM_007294	exon10	c.3448C>T	p.Pro1150Ser
F28	B00875	*MSH2*	NM_000251	exon13	c.2055A>G	p.Ile685Met
F28	B00876	*BRCA1*	NM_007294	exon10	c.3448C>T	p.Pro1150Ser
F28	B00878	*BRCA1*	NM_007294	exon10	c.4084_4085del	p.Asp1362fs
F28	B00878	*BRCA1*	NM_007294	exon10	c.3448C>T	p.Pro1150Ser
F28	B00879	*BRCA1*	NM_007294	exon10	c.3448C>T	p.Pro1150Ser
F28	B00880	*BRCA1*	NM_007294	exon10	c.3448C>T	p.Pro1150Ser
F28	B00887	*MSH2*	NM_000251	exon13	c.2055A>G	p.Ile685Met
F28	B00888	*BRCA1*	NM_007294	exon10	c.4084_4085del	p.Asp1362fs
F28	B00888	*MSH2*	NM_000251	exon13	c.2055A>G	p.Ile685Met
F28	B00889	*BRCA1*	NM_007294	exon10	c.4084_4085del	p.Asp1362fs
F28	B00889	*MSH2*	NM_000251	exon13	c.2055A>G	p.Ile685Met
F29	B00883	*RAD50*	NM_005732	exon21	c.3310T>C	p.Tyr1104His
F29	B00884	*MSH6*	NM_000179	exon4	c.2522delG	p.Arg841fs
F29	B00884	*RAD50*	NM_005732	exon21	c.3310T>C	p.Tyr1104His
F29	B00905	*MSH6*	NM_000179	exon4	c.2522delG	p.Arg841fs
F29	B00905	*RAD50*	NM_005732	exon21	c.3310T>C	p.Tyr1104His
